# Reelin controls the positioning of brainstem serotonergic raphe neurons

**DOI:** 10.1371/journal.pone.0200268

**Published:** 2018-07-12

**Authors:** Reham Shehabeldin, David Lutz, Meliha Karsak, Michael Frotscher, Kerstin Krieglstein, Ahmed Sharaf

**Affiliations:** 1 Institute of Anatomy and Cell Biology, Department of Molecular Embryology, Albert-Ludwigs-Universität, Freiburg, Germany; 2 Institute for Structural Neurobiology, Center for Molecular Neurobiology Hamburg (ZMNH), University Medical Center Hamburg-Eppendorf (UKE), Hamburg, Germany; 3 Research Group Neuronal and Cellular Signal Transduction, ZMNH, University Medical Center Hamburg-Eppendorf (UKE), Hamburg, Germany; 4 Department of Histology and Cytology, Faculty of Veterinary Medicine, Zagazig University, Zagazig, Egypt; Radboud University Medical Centre, NETHERLANDS

## Abstract

Serotonin (5-HT) acts as both a morphogenetic factor during early embryonic development and a neuromodulator of circuit plasticity in the mature brain. Dysregulation of serotonin signaling during critical periods is involved in developmental neurological disorders, such as schizophrenia and autism. In this study we focused on the consequences of defect *reelin* signaling for the development of the brainstem serotonergic raphe system. We observed that *reelin* signaling components are expressed by serotonergic neurons during the critical period of their lateral migration. Further, we found that *reelin* signaling is important for the normal migration of rostral, but not caudal hindbrain raphe nuclei and that *reelin* deficiency results in the malformation of the paramedian raphe nucleus and the lateral wings of the dorsal raphe nuclei. Additionally, we showed that serotonergic neurons projections to laminated brain structures were severely altered. With this study, we propose that the perturbation of canonical *reelin* signaling interferes with the orientation of tangentially, but not radially, migrating brainstem 5-HT neurons. Our results open the window for further studies on the interaction of *reelin* and serotonin and the pathogenesis of neurodevelopmental disorders.

## Introduction

The serotonergic (5-HT) system is involved in the development of the central nervous system (CNS) and modifies adult behavior and the fine wiring of brain connections [1]; [2,3]; [4]; [5]. Serotonin might act as a growth regulator during early brain development prior to its role as a neurotransmitter in the adult brain. Such a function appears likely for two reasons: First, there is a regional expression of serotonin receptors early in embryonic development. Second, maternal serotonin may reach the fetus via the placenta [6]. Indeed, the serotonergic system has been implicated in myriad developmental events such as cell proliferation, migration, neuronal differentiation and synaptic plasticity [1];[7]. Serotonergic neurons represent one class of early born and widely distributed neuronal cells in the mammalian brain. They are born near the floor plate at embryonic day (E) 10–11.5 in the mouse. These neurons collectively form the raphe system and cluster in distinct nuclei (B1-B9). [8]described two steps in the migration of hindbrain serotonergic neurons to their final destinations: Initial migration from the ventricular zone along the midline during early embryonic development, followed by a secondary migration wave extending into postnatal life. The specification of hindbrain serotonergic neurons is controlled by different growth factors including sonic hedgehog (Shh) and FGF, especially Fgf8 and Fgf4 together with three main transcription factors (Nkx2.2, Pet1 and Gata3a), which direct conversion of 5-HT precursors to 5-HT postmitotic precursors and then to fully differentiated 5-HT neurons [9–12]. The serotonergic system provides widespread projections to the CNS [13–15].The rostral serotonergic raphe neurons extend their axonal arborizations to the midbrain, forebrain as well as to the cerebellum [16–17].The full maturation of serotonergic neurons continues through the third postnatal week [18]. In rodents, the serotonergic projections to the neocortex develop very early at E15-E17 and establish synaptic contacts with Cajal-Retzius cells, which are known to secrete reelin.

Reelin is a multifunctional extracellular matrix protein that controls diverse aspects of normal brain development and function. Deficient reelin signaling does not only result in neuronal migration defects and malpositioning of cortical, hippocampal and cerebellar neurons [19–24], but also has consequences for the positioning of brainstem cells such as mesencephalic dopaminergic neurons [25–27] and hindbrain motor neurons [28]. Pharmacological manipulation of the serotonergic system during very early brain development altered reelin expression levels [29] which might explain indirect effects of the serotonergic system on the late stages of embryonic cortical development mediated by reelin [30–32].There is evidence that the serotonergic systems as well as reelin levels are dysregulated in several neurological disorders such as autism and schizophrenia[33–38]. Reelin signals via binding to apolipoprotein E receptor 2 (ApoER2) and the Very Low Density Lipoprotein Receptor (VLDLR) on migrating neurons and/or radial glial cells, which transmit the reelin signal via Src family kinases[24, 39, 40]. Mice deficient in both ApoER2 and VLDLR or Disabled 1 (Dab1) display a reeler-like phenotype [39, 41, 42]. Postnatally, reelin plays an important role in synaptic plasticity and connectivity [43–49]. Serotonin activity controls the overall length of dendrites and the formation of dendritic spines [50–53]. Here we used different mutant mice to study a potential role of reelin signaling in the development of the serotonergic raphe system, with particular focus on the normal organization, migration, and projection of rostral raphe nuclei.

## Materials and methods

### Animals

Mice were housed in standard cages and maintained on a 12/12h light/dark cycle at 25°C, with food and water ad libitum. Animals were purchased from the Jackson Laboratory (Bar Harbor, ME, USA). All animal experiments were approved by the Ethic Committee of the University of Hamburg (license no. Org_604) and Freiburg University conform to the guidelines set by the European Union. (Permit number: X-11/18H). Animals were sacrificed under xylazin and ketamine anaesthesia and all efforts were made to minimize suffering. Generation, breeding and genotyping of reeler and Dab1 mutants were performed as described previously [54,55]. The ApoER2/VLDLR double knockout mice were all of a VLDLR-/- genetic background [56]. The CXCR4-eGFP mice were obtained from Dr. Max Anstötz, Department of Neuroanatomy, Unversity Medical Center, Hamburg [57]. The day of the vaginal plug was recorded as embryonic day 0.5 (E 0.5). The first neonatal day was considered to be postnatal day 0 (P0).

### Immunohistochemistry

For histological and immunohistochemical analysis animals were anesthetized then perfused transcardially with 4% paraformaldehyde (PFA). Brains were dissected, post-fixed in 4% PFA and sectioned into 50μm thick vibratome sections (Leica Microsystems). However for serotonin transporter protein (SERT) staining, we prepared cryo-sections 20μm thick, in addition the cryo-sections exposed to antigen retrieval using citrate buffer PH = 6. Immunohistochemistry was performed following standard protocols. Briefly, immunohistochemical procedures were carried out on free floating and cryo-sections using primary rabbit anti-5HT antibody (1:500, Sigma-Aldrich), mouse anti-vimentin antibody (1:300, Sigma-Aldrich, Catalog number. V5255); mouse anti-caspase-3 (1:250, Abcam), rabbit anti-Dab1 antibody (1:500, Abcam, ab111684)), mouse G10 (anti-reelin) antibody (1:400, Millipore, MAB5364), rabbit anti-SERT (Alomone labs, 1:300), Goat anti-Serotonin antibody (Abcam #66047, 1:500) and rabbit anti-VLDLR antibody (1:200, Santa Cruz Biotechnology, sc-20746). Blocking of non-specific binding sites was performed using blocking solution (phosphate buffered saline (PBS) + 10% of 0.1% Triton X-100 (Sigma, Germany) + 10% normal goat serum (Gibco) at room temperature for 1 hour). Subsequently, sections were washed in PBS and incubated with goat anti-mouse Alexa Fluor 568 (1:500) and goat anti-rabbit Alexa Fluor 488 (1:500) secondary antibodies (Molecular Probes) for 1 hour at room temperature. Then, the sections were incubated in DAPI in PBS for 10 minutes, mounted using Fluoromount (Fluka), and stored in the dark at 4°C. Images were taken using a Keyence Fluorescence Microscope (BZ-9000, Keyence) and processed with ImageJ software.

Numbers of 5-HT-labeled neurons were counted on 40 μm thick serial coronal brain stem sections, the 5-HT positive neurons which have darkly stained cytoplasm and clearly visible unstained nuclei were only counted using the Keyence microscope software. We counted all the sections throughout the rostro-caudal axis of the brainstem of reeler mutants; Dab1 mutants, and ApoER2/VLDLR double knockout mice.

### Western blotting analysis

The brain protein lysates such as cortex, hippocampus, and cerebellum were collected from both wild type and reeler mutants (n = 3) mixed with 0.2% DDM lysis buffer consisting of (6× Laemmli buffer containing 100 mM Tris–HCl (pH 6.8), 4% SDS, 60% glycerol, 0.2% bromophenol blue and 10 mM DTT in dH2O). Proteins of the cell lysates were separated by12% SDS-PAGE gels with constant voltage of 110 V for 75 min and transferred to Amersham Hybond ECL nitrocellulose membrane (RPN2032D, GE Healthcare, Germany). After blocking with 5% skimmed milk powder in TTBS (150 mM NaCl, 10 mM Tris and 0.025% Tween® 20 in dH2O), the membrane was incubated with rabbit anti-SERT and anti-ß-actin primary antibodies in TTBS or blocking buffer over night at 4°C. After three washing steps with TTBS the membrane was incubated with rabbit fluorescent infra-red dye-conjugated antibodies (800 and 680nm) for 1h at RT. The membrane was washed for three times in TTBS and immunoreactive proteins were visualized with the LI-COR Odyssey® imaging system Germany.

### Statistical analysis

Statistical analysis was performed using Student's unpaired t-test, the data are presented as mean ± SD. Differences were considered statistically significant at ***p<0.001.

## Results

Our study was aimed at analyzing a potential role of reelin signaling during the pre- and postnatal development of brainstem serotonergic neurons.

### Normal organization of the rostral brainstem serotonergic raphe neurons in wild type mice

**Fig 1:**
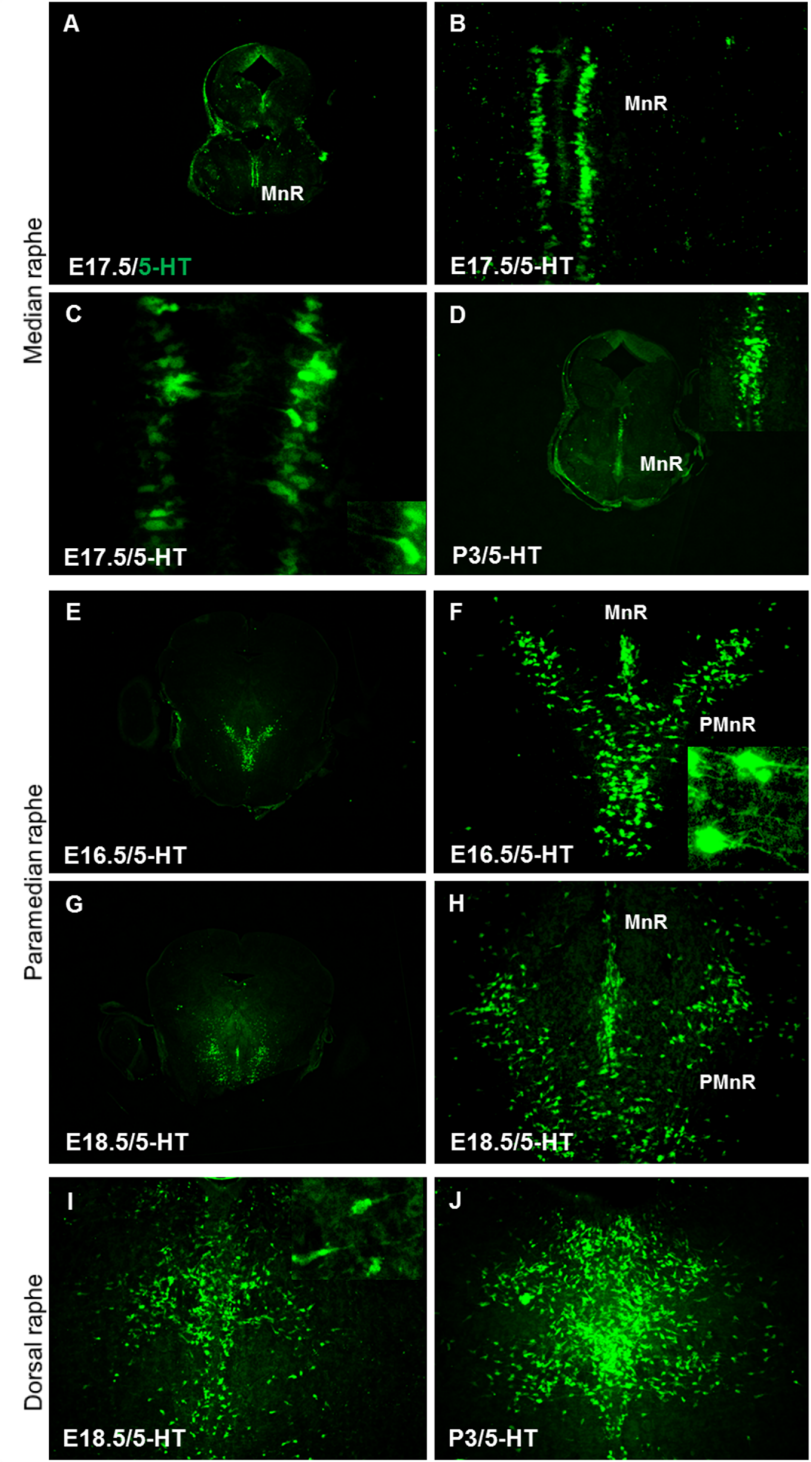
Normal organization of serotonergic neurons in the brainstem of wild type mice. (A) 5-HT-positive neurons of median raphe extend along the midline of the brainstem at E 17.5. (B) Higher magnification of the boxed area in (A) Showing median raphe neurons arranged in two parallel columns C. Higher magnification of boxed area in B showing the orientation of leading processes of median raphe neurons (arrow heads, inset in C). (D) Midline fusion of median raphe neurons is observed at postnatal day 3 (P3, inset in D). (E) The paramedian raphe neurons (PMnR) arranged laterally to the median raphe neurons on both sides of the midline of the brainstem at E16.5. (F) The future paramedian raphe neurons extend their leading processes laterally (inset). (G) The paramedian raphe neurons separated from median raphe at E18.5. (I) The dorsal raphe neurons (DRN) migrate ventrally from the ventricular zone at E18.5. Inset in I showing higher magnification of the boxed area reveals that neurons of DRN extend their leading processes laterally and migrate toward the pial surface. MnR, median raphe; PMnR, paramedian raphe; DRN, Dorsal raphe neurons. Scale bars in A, D, E, and G: 300 μm; all other panels: 100μm.

To characterize the migratory routes of rostral brainstem serotonergic raphe neurons, we used 5-HT immunohistochemistry to label postmitotic serotonergic neurons at different embryonic time points. In wild type embryos at E17.5, we found that the serotonergic neurons of median raphe (MnR, B8) were bilaterally oriented along the midline, running in parallel to the dorso-ventral axis of the brainstem and migrating along radial glial processes, which resulted in the formation of two cell columns (Fig 1A–1C). Later on, the cells of the two columns extended their leading processes toward the midline of the brainstem (Fig 1C and inset) and finally underwent a complete midline fusion, forming the median raphe nucleus at P3 (Fig 1D and inset). The paramedian raphe neurons (PMnR, B9) migrate also ventrally along radial glia cells and settle together with the median raphe neurons along the midline of the brainstem at E15.5-E16.5 (Fig 1E and 1F). However, the future paramedian raphe neurons extend their leading processes laterally (Fig 1F, inset) and then migrate into the ventrolateral tegmentum of the pons and midbrain to finally organize on both sides of the median raphe nearby the pial surface at E18.5 (Fig 1G and 1H). PMnR neurons do not fuse and remain as bilateral nuclei in a ventral position to the reticular formation. The neurons of the dorsal raphe (DRN) migrate ventrally from the ventricular zone and migrate for a short distance along the midline of the brainstem around E18.5. The neurons then extend their leading processes laterally and migrate toward the pial surface (Fig 1I and inset) to finally form the lateral wings of the dorsal raphe at P3 (Fig 1J). The pontine raphe nucleus (B5) neurons migrate to settle on both sides of the midline parallel to the dorsoventral axis of the brainstem forming two columns. Later on, these neurons extend their leading processes toward the midline and eventually the cells on both sides fuse.

### Localization patterns of reelin and its downstream signaling components in brainstem serotonergic neurons during embryonic development

**Fig 2:**
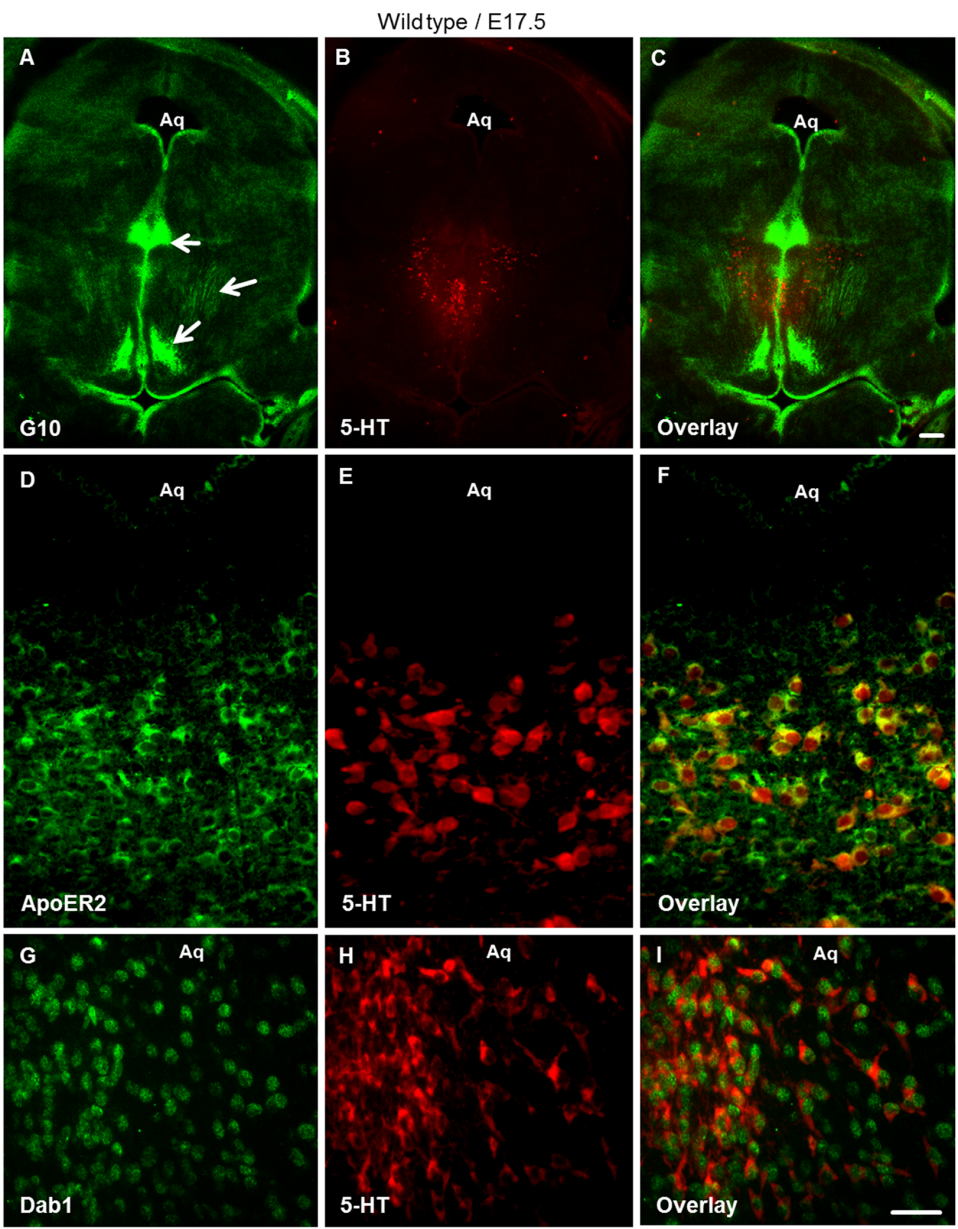
Reelin signaling components expressed in the brainstem of wild type embryos at E17.5. (A-C) Double immunostaining for G10 (anti-reelin antibody, green) and anti-5-HT (red) revealed positive reelin immunoreactivity in dorsolateral as well as ventral brainstem regions (arrows). (D-F) Showed Immunostaining for ApoER2 (green) and 5-HT (red) and (G-I) Revealed immunostaining for anti-Dab1 (green) and 5-HT (red) on vibratome brainstem slices at E17.5. Aq. Aqueduct. Scale bar A-C: 300μm; D-I: 100μm.

In order to learn more about the potential functions of the reelin signaling pathway in the brainstem and its role in the normal development of the serotonergic raphe system, we first analyzed the spatiotemporal presence of reelin signaling components during early brainstem development. At E17.5, the critical time point for the lateral migration of rostral serotonergic neurons, immunohistochemistry was performed on vibratome sections through the brainstem using antibodies against reelin (G10) and 5-HT, the late marker of serotonergic neurons. We observed that reelin is expressed in specific neuronal populations around the midline as well as in lateroventral regions of the brainstem (Fig 2A–2C). Moreover co-staining for reelin receptors using anti-ApoER2 together with 5-HT antibodies on brainstem sections revealed that ApoER2 is expressed in 5-HT neurons during the critical period of lateral migration at E17.5 (Fig 2D, 2E and 2F). Similarly, VLDLR is also expressed by 5-HT positive neurons at E17.5 (S1 Fig). In addition, disabled-1 (Dab1), an adaptor protein interacting with the reelin receptors, was expressed at E17.5 in serotonin-positive neurons (Fig 2G, 2H and 2I). Reelin and Dab1 expression were considerably weaker in the brainstem at E13.5 and E15.5 as compared with E17.5 (S2 Fig). We conclude from these results that reelin signaling pathway components are present in the raphe nuclei and may have a potential impact on the normal development of brainstem serotonergic neurons.

### Altered distribution of serotonergic neurons in reeler mutants is restricted to rostral raphe nuclei

**Fig 3:**
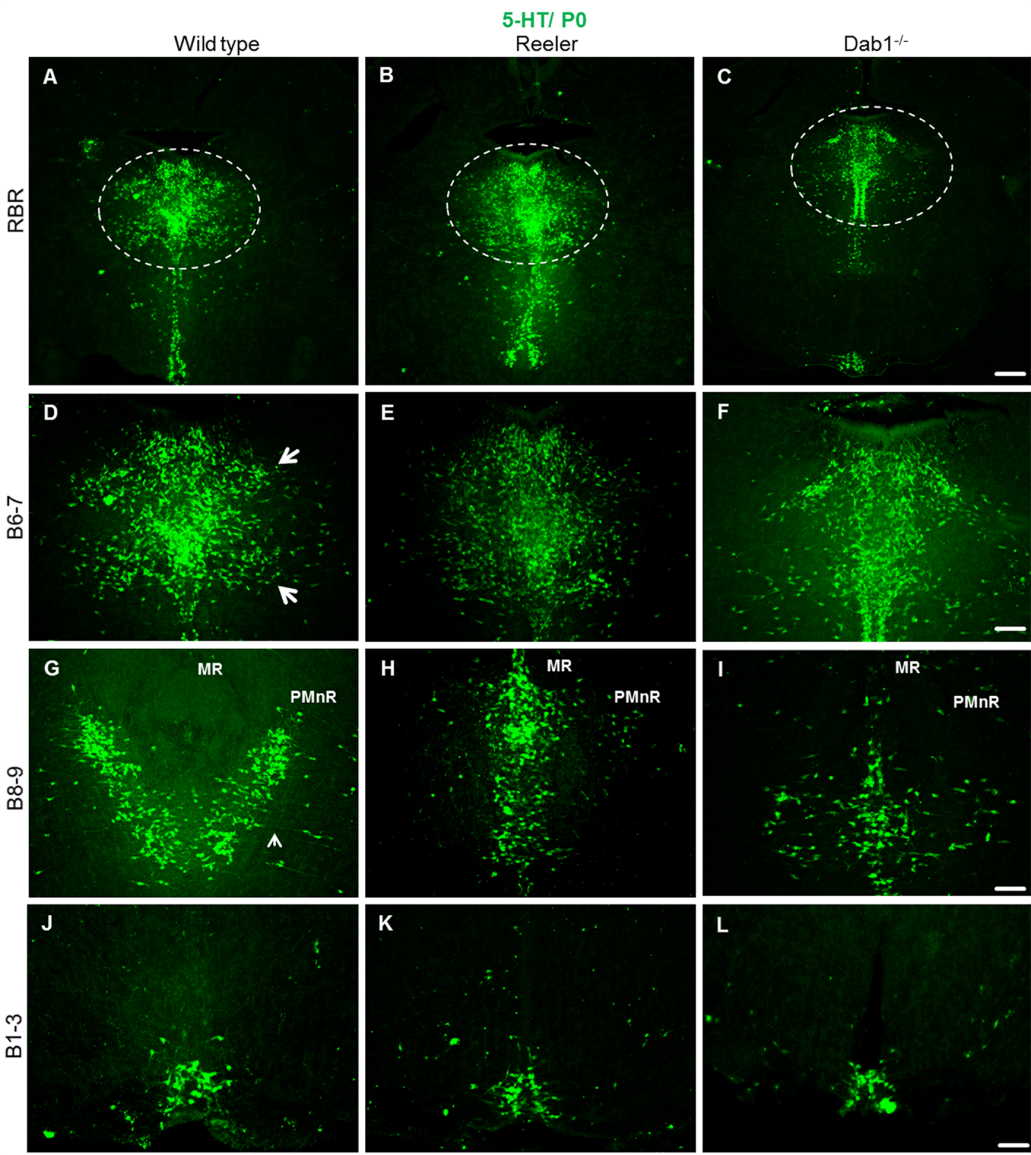
Altered distribution of rostral brainstem serotonergic raphe nuclei but not caudal ones in reeler and Dab1- deficient mice at P0. Coronal sections of wild type (A-J), reeler mutant (B-K) and Dab1^-/-^ (C-L) Showing the serotonergic nuclei throughout the rostro-caudal extent of the brainstem. rostral brainstem raphe (RBR) neurons in the wild type (A), reeler (B), and Dab1 mutants (C). (D) Normal formation of the lateral wings of the dorsal raphe (arrows, B7) in wild type. (E, F) lateral wings on both sides of the dorsal raphe nuclei have not formed properly in both reeler and Dab1 mutants. (G) Showing the laterally distributed PMnR on both sides of the median raphe in the ventrolateral brainstem region of wild type mice. (H, I) Note poor development of PMnR in reeler and Dab1 mutants. 5-HT-positive neurons destined to PMnR accumulate together with MnR neurons in the midline of brainstem. (J-L) There are no obvious differences between genotypes in the caudal raphe neurons (B1-B3). Scale bar A-L: 100μm.

**Fig 4:**
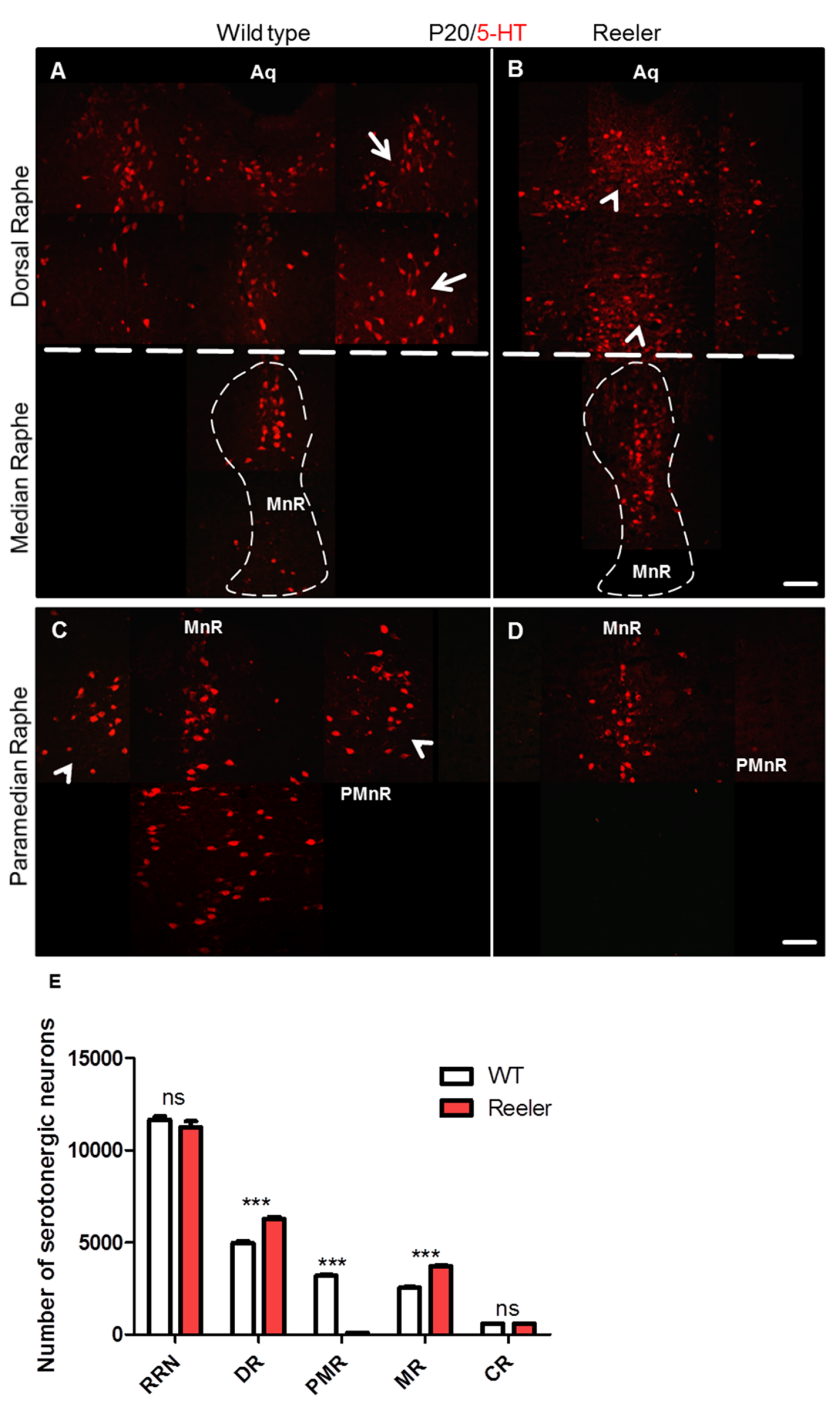
Loss of paramedian raphe neurons and the lateral wings of the dorsal raphe in reeler mutants at P20. (A) Normally organized lateral wings of the dorsal raphe nuclei (white arrows) as well as median raphe in wild type mice (dashed line). (B) In reeler mutants the lateral wings of the dorsal raphe nuclei are not formed properly and 5-HT positive cells were accumulated in the dorsal raphe body (arrowheads). Moreover, relatively more 5-HT positive neurons are observed in the median raphe (dashed line) as compared with the wild type embryos. (C) Photomicrograph shows that the paramedian raphe neurons in a wild type section (arrow heads) are well organized on the both sides of the median raphe in the venrolateral region of the brainstem. (D) No formation of the paramedian raphe in reeler mutants. (E-G) Show significant reduction in the number of 5-HT- positive neurons of paramedian raphe and in turn higher neuron numbers in the median as well as dorsal raphe neurons in reeler mutants as compared to wild type mice. Thus, the total number of rostral raphe neurons was not significantly different between wild type animals and reeler mutants. Data are presented as means values ± S.E.M from 4 independent animals. Differences between groups are shown ***P<0.001. Aq. Aqueduct. Scale bar A-D: 100μm.

Studies of serotonergic neurons born in the subventricular zone as early as on E 10 and migrating from the ventricular zone until they arrest and form the raphe nuclei of the brainstem and pons have been hindered by the lack of early markers specific for these neurons. To investigate the role of reelin signaling during the normal organization of serotonergic neurons during early development, reeler mice, Dab1-deficient as well as ApoER2- and VLDLR-deficient double knockout (DKO) embryos were collected at P0. Coronal brainstem vibratome sections were cut and immunostained with an antibody against 5-HT. In wild type embryos we found that the hindbrain serotonergic nuclei were clearly identifiable and located in their definitive positions (Fig 3A, 3D, 3G and 3J). In contrast, in reeler (Fig 3B, 3E, 3H and 3K) as well as in Dab1 knockout mice (Fig 3C, 3F, 3I and 3L) two cell populations derived from the same site of origin were severely misplaced, which has not been noticed in previous studies on mice with defect reelin signaling. These two cell populations were the dorsal raphe (DR, B7) and the paramedian raphe (PMnR, B9, also called supralemniscal raphe) nuclei. In wild type mice the PMnR is located ventrolaterally in the brainstem (rhombomeres 1–3) [58], lateral to the median raphe, extending into the mesencephalic reticular formation. In wild type embryos, the position of the PMnR remains stable during different postnatal stages (P0 and P20; see Figs 3G and 4C). In reeler mutants, the paramedian raphe neurons appear to be absent on both sides at these stages (Figs 3H and 4D). In contrast, relatively more 5-HT-positive neurons have accumulated along the midline (together with median raphe neurons and neurons of the dorsal raphe neurons (DRN) (Fig 3H). In Dab1 mutants, very few cells were still able to migrate laterally and reach their normal destinations (Fig 3I). Moreover, we found the lateral wings of DRN (DR, B7) in reeler mutants and Dab1 knockout mice very much reduced at P0, and relatively more serotonin-positive cells accumulated along the midline. As a result, the lateral wings of the dorsal raphe failed to form the characteristic symmetric cell accumulations of wild type animals (See Fig 3E and 3F and Fig 4A and 4B). We conclude that in mice deficient in reelin/Dab1 signaling both the paramedian and dorsal raphe neurons were severely misplaced. In contrast, caudal hindbrain serotonergic neurons (B1-B3) in both wild type and reeler mutants as well as in Dab1 knockout mice appeared very similar (Fig 3J–3L). Moreover, the results suggest that the phenotype of brainstem 5-HT neurons observed in both reeler and Dab1 mutants is due to a defect in lateral migration rather than to apoptotic cell death since our cell counts revealed a significant increase in 5-HT-positive neurons in MR and DR on expense of cells in the PMnR (Fig 4E).

### A reeler-like phenotype is observed in apoer2/vldlr double knockout mice

**Fig 5:**
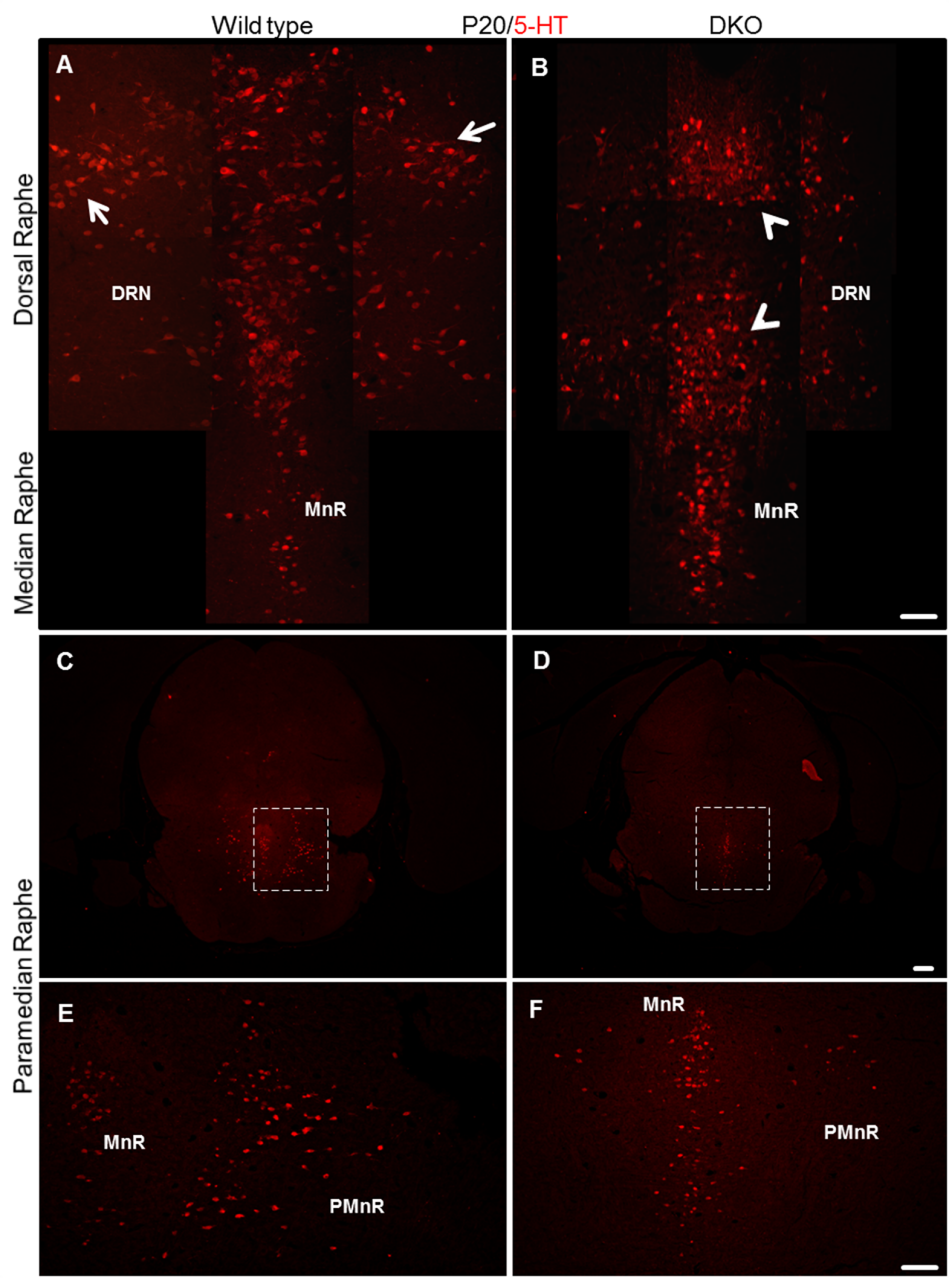
Reeler-like phenotype observed in apoer2/vldlr doube knockout mice (DKO) at P20. (A) Normal formation of the two lateral wings (arrows) of the dorsal raphe nuclei in wild type. (B) In DKO mice, the lateral wings of the dorsal raphe nuclei do not develop properly. In turn, more 5-HT neurons accumulate in the midline (arrowheads) as compared to wild type. (C) Normal development of the paramedian raphe on both sides of the median raphe in a wild type section at P20 (see boxed area shown at higher magnification in E). (D) Striking loss of the paramedian raphe in a DKO mouse at P20 as compared to an aged-matched wild type mouse (see boxed area shown at higher magnification in F). Scale bar A, B, E, and F: 100μm; C, D: 300μm.

We have described previously that the canonical reelin signaling pathway is required for the normal positioning of mesencephalic dopaminergic neurons [25]. To investigate whether the phenotype observed in the serotonergic system of reeler and Dab1 knockout mice is mediated via the reelin receptors ApoER2 and VLDLR, we next studied the organization of brainstem serotonergic neurons in mice deficient in both apoer2 and vldlr (DKO) at P20, again by immunohistochemical staining of coronal brainstem vibratome sections using the 5-HT antibody. We found that the paramedian raphe nuclei are totally lost and the lateral wings of the dorsal raphe are not properly formed, similar to the phenotype that we had observed in reeler mutants. Moreover, many more 5-HT positive neurons accumulated in the body of the dorsal raphe and in the median raphe in this double knockout (DKO) mice when compared with wild type sections (Fig 5A–5F). As there is no specific marker available to differentiate between different raphe neurons of the brainstem, we were not able to identify the misplaced cells in the dorsal and median raphe. Cell counts performed by a trained person blinded to the experimental conditions revealed a significant increase in the total number of neurons in the median and dorsal raphe as well as a striking reduction of paramedian raphe neurons, reminiscent of our findings in reeler mutants. We concluded that the reduction of the paramedian raphe was due to defect lateral migration of the respective cells. Furthermore, when we performed co-immunostaining using caspase-3 as a marker of apoptotic cells, we failed to detect an increase in apoptotic cells, neither in reeler nor in Dab1 mutants and DKO embryos. Together the results suggest that the severe reduction of the paramedian raphe and of the lateral wings of the dorsal raphe was due to a migration defect rather than increased cell death.

### The formation of radial glial fibers is not different between reeler and wild type animals at E17.5

**Fig 6:**
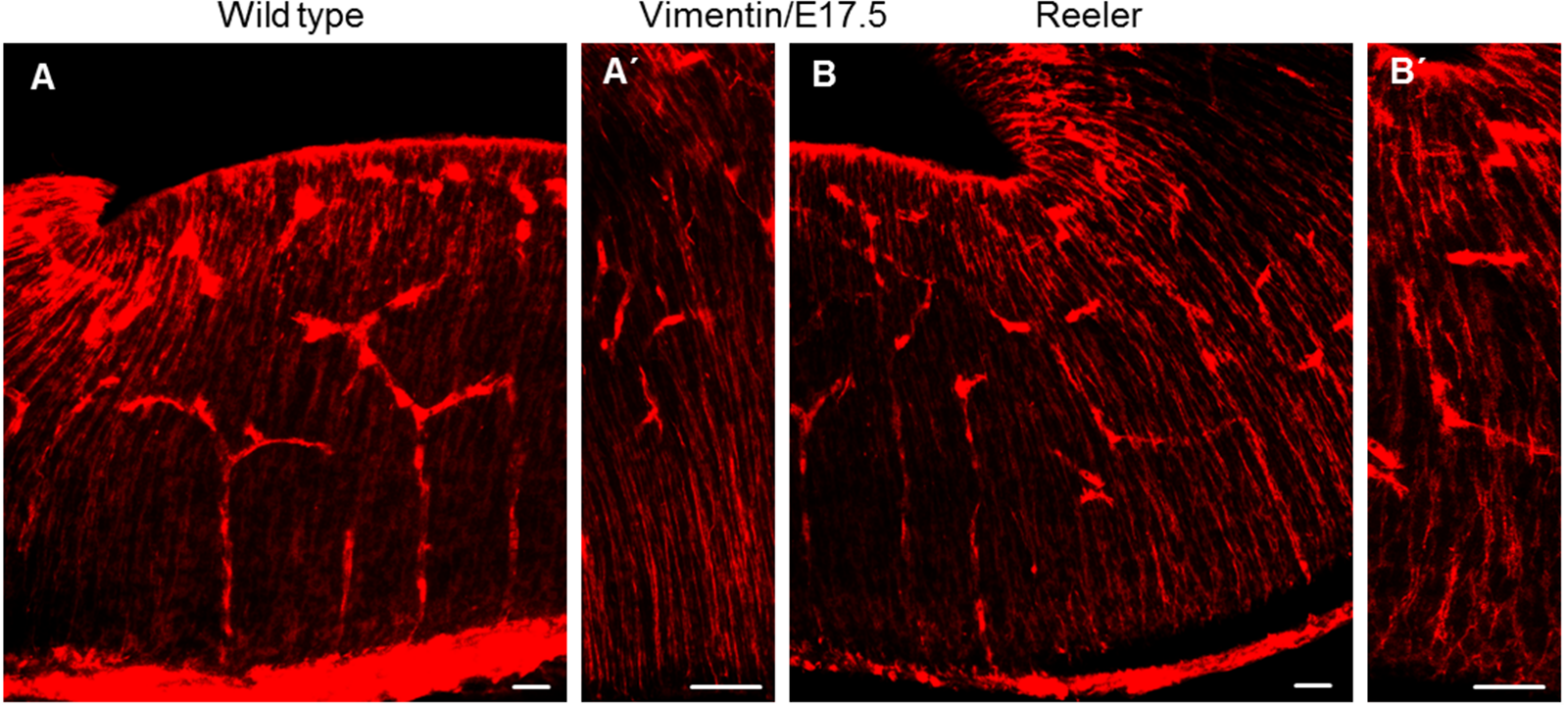
Normal radial glia development in reeler mutant at E17.5. Immunostaining for vimentin to label radial glial cells on vibratome sections of the ventral brainstem. Vimentin staining was comparable between wild type (A, A’) and reeler sections (B, B’). Scale bars: 100μm.

The most affected nuclei in reelin- or Dab1-deficient mice are the paramedian raphe and the lateral wings of the dorsal raphe nuclei. The 5-HT positive neurons of both paramedian raphe and dorsal raphe migrate ventrally, probably using radial glial fibers as a scaffold, and then laterally until they reach their final location in the lateral brainstem. Using immunostaining against vimentin, a marker for radial glia cells, on vibratome sections at the level of rhombomeres 1, 2 and 3 of the brainstem in both wild type and reeler embryos at embryonic day 17.5 did not provide evidence of abnormal fibers, neither with respect to process length nor end-feet attachment (Fig 6A, 6A’, 6B and 6B’). This result might explain why the radial migration of serotonin- positive neurons of the dorsal and paramedian raphe was not affected in reeler mutants. However, the lateral migration of the lateral wing neurons and the neurons of the paramedian raphe was arrested, resulting in a cell accumulation along the dorsoventral axis of the brainstem, thus enlarging the dorsal and median raphe nuclei.

### Altered serotonergic projections in reeler mutants

**Fig 7:**
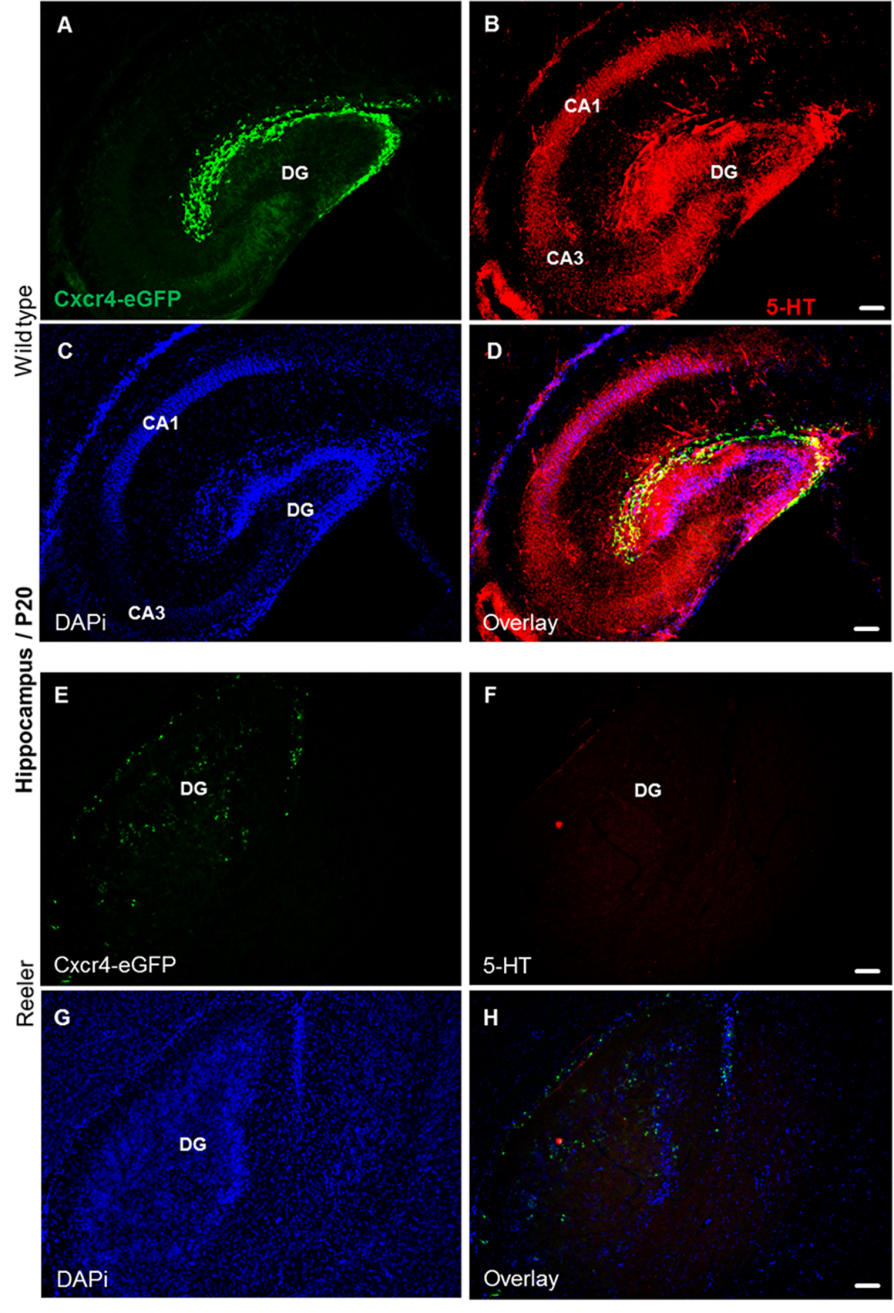
Altered serotonergic innervation of the reeler hippocampus at P20. (A) Expression of Cxcr4-eGFP in Cajal-Retzius (CR) cells of the dentate gyrus. (B-D) Serotonergic fibers are distributed throughout hippocampal layers, and 5-HT positive fibers and Cajal-Retzius cells overlap. (E) Scattered distribution of Cajal-Retzius cells in reeler hippocampus. (F-H) Severe reduction of serotonergic fibers in Cxcr4-eGFP hippocampal reeler mice. CA1, cornu ammonis area 1; CA3, cornu ammonis area 3; DG, dentate gyrus. Scale bar for A-D: 100μm.

**Fig 8:**
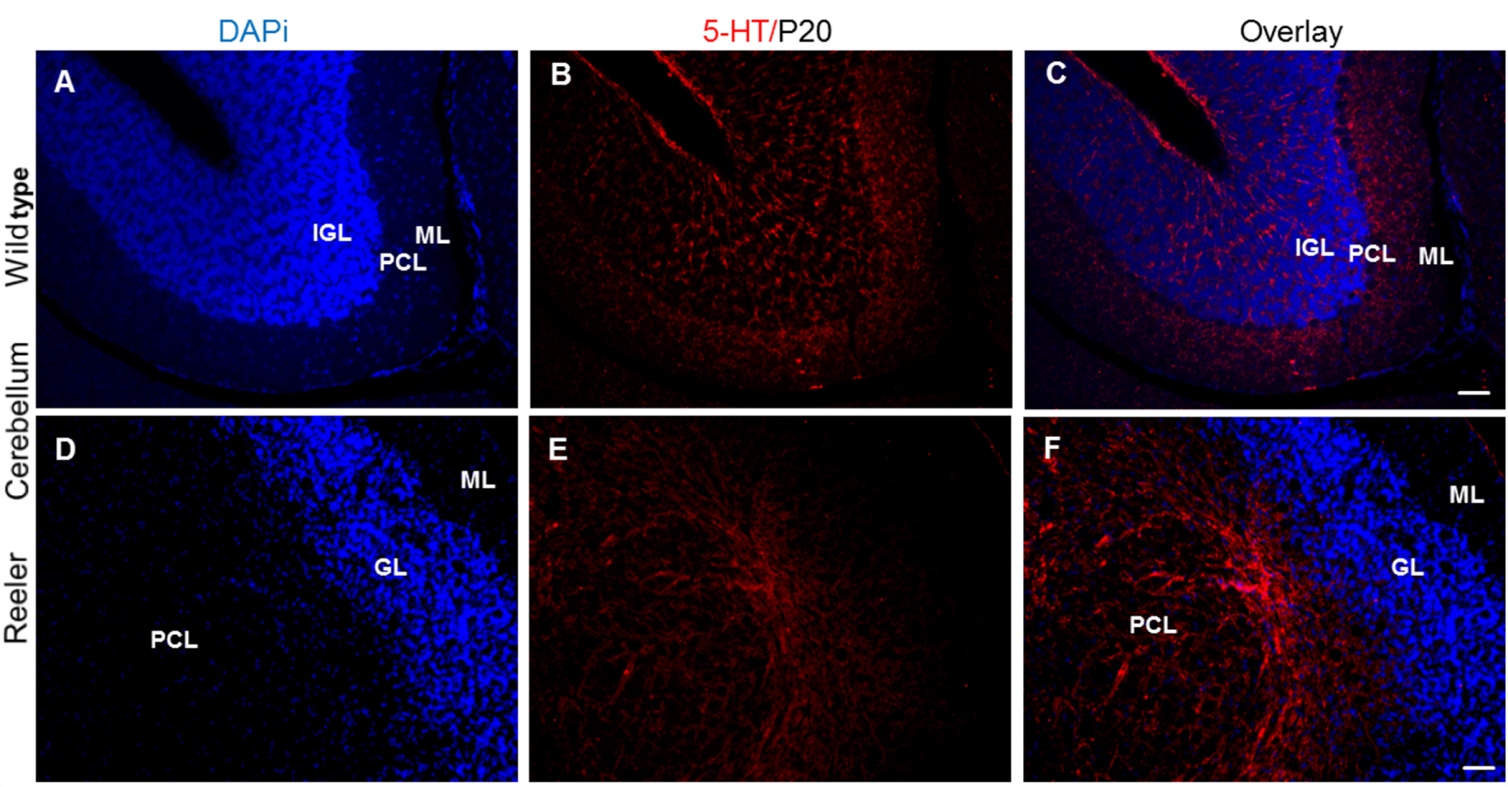
Altered serotonergic innervation of cerebellum in reeler mutants at P20. (A-C) 5-HT positive fibers in the wild type cerebellum extend into the granule cell layer and Purkinje cell layer as well as to the outer molecular layer. (D-F) 5-HT positive fibers in the reeler cerebellum extend only to Purkinje cell layer, which are aberrantly located in the cerebellar white matter and 5-HT fibers do not reach to reeler granule cells and outer molecular cell layer. PCL. Purkinje cell layer; IGL. Inner granular layer; OML. Outer molecular layer; GL. Granule cell layer. Scale bar A-D: 100μm.

The proper functioning of the serotonergic system requires the coordinated formation of projections extending from brainstem serotonergic raphe neurons to a wide range of target regions, including the prefrontal cortex, hippocampus, and cerebellum. In wild type at P20, we found that the hippocampal regions, dentate gyrus (DG), cornu ammonis area 1 (CA1), cornu ammonis area 3 (CA3), dentate gyrus (DG) receive a dense 5-HT positive innervation with distinct laminar patterns (Fig 7A–7D). The serotonergic fibers innervate the molecular layer, granule cell layer and hilus of the dentate gyrus (DG), thus also reaching Cajal-Retzius cells that are still present in the outer molecular layer of the DG at this stage. A particularly dense plexus of serotonergic fibers terminate in the cell layers, which is largely lost and the cells are dispersed in reeler, as seen when compared to wild type mice. As a result, also the dense serotonergic innervation of cell layers is lost (Fig 7E–7H). In the wild type cerebellum, serotonergic axon terminals run in between the cell bodies of the inner granular layer and also surround the somata of Purkinje cells; some fibers extend as far as to the outer molecular layer (Fig 8A–8C). Thus, they can establish synaptic connections with a variety of cell types. In contrast, the serotonergic projections were severely altered in the reeler cerebellum lacking its normal cytoarchitectonic organization (Fig 8D). In reeler cerebella, 5-HT axons extend to misplaced Purkinje cells, which are abnormally located in the cerebellar white matter. No 5-HT-positive fibers could be seen around the granular cells and in the outermost molecular layer (Fig 8E and 8F).

**Fig 9:**
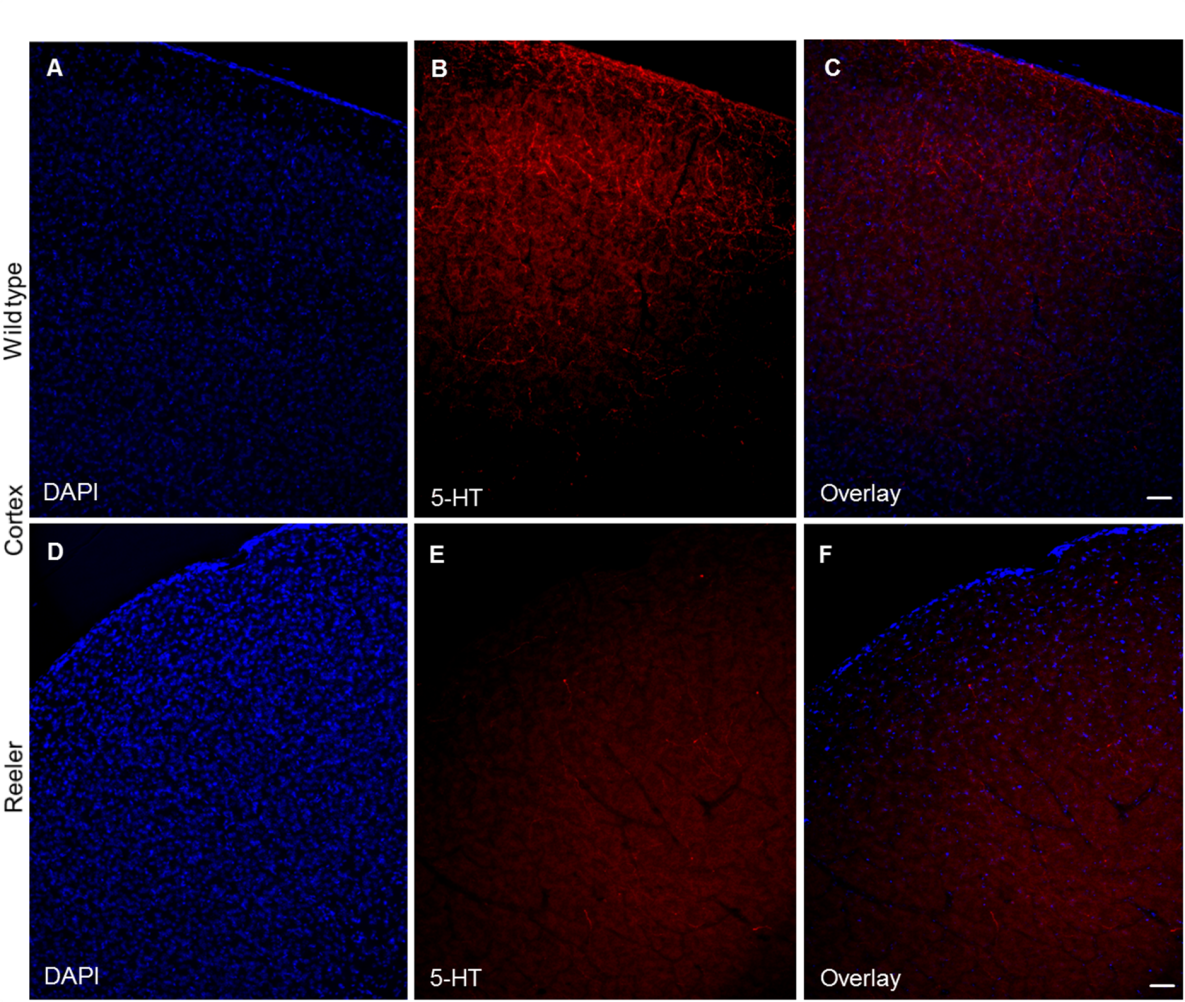
Altered serotonergic innervation in reeler cortex at P20. (A-C) Dense projections of 5-HT positive fibers to all cortical layers in the wild type mice. (D-F) Show severe reduction of 5-HT positive serotonergic fibers in cortical layers in reeler mutants. Scale bars for A-F: 100μm.

**Fig 10:**
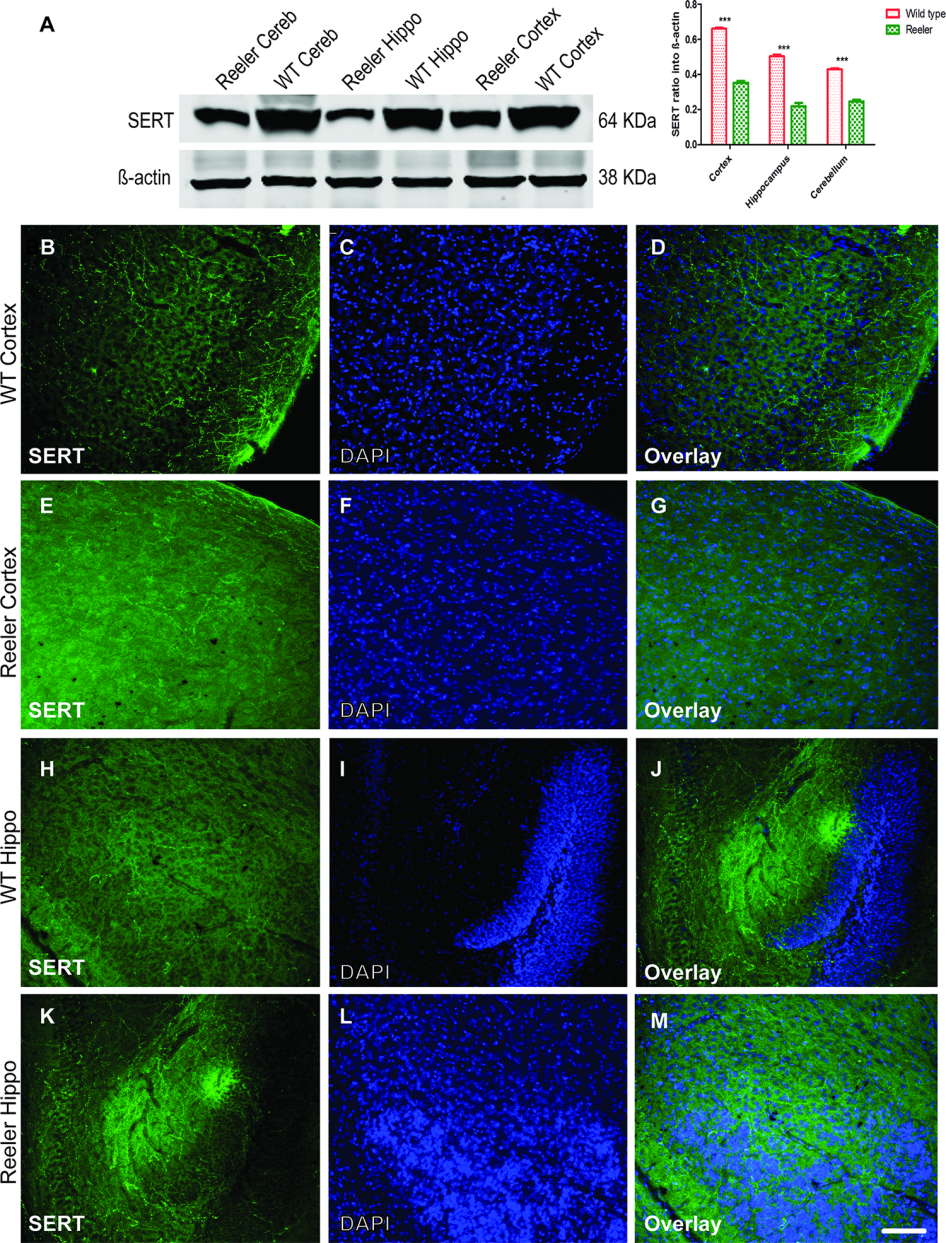
Reduction of serotonin transporter protein (SERT) expression in brain regions of reeler mice. A. Western blot analysis showed reduction of SERT protein levels in reeler cortex, hippocampus, and Cerebellum as compared to wild type. (B-G) reduction of SERT expression in reeler cortex as well as in hippocampal slices as observed in (H-M) as compared to the same matched wild type littermates at P30. Hippo = hippocampus; Cereb = Cerebellum. Scale bar: 100 μm.

The cerebral cortex of wild type animals shows a diffuse innervation by serotonergic fibers in the form of a superficial and deep plexuses (Fig 9A–9C). In reeler, a reduction of the serotonergic projections was observed, which was particularly obvious for the superficial plexus (Fig 9D–9F). By western blot and immunostaining analysis antibody against SERT, a selective re-uptake system, which transports released serotonin back into serotonergic neurons. We observed that SERT is highly expressed in the protein lysates collected from cortex, hippocampus and cerebellum of wild type as compared to reeler mutants postnatal day 30 (P30) (Fig 10A). The SERT protein levels by western blot were decreased in reeler by at least 50% from control wild type in some brain regions. In addition to the western blot results, we conducted also immunostaining using anti-SERT antibody gave the same conclusion like that observed from the localization pattern of anti-5HT antibody in the laminated brain structures (Cortex, Hippocampus, and cerebellum). The results showed that SERT protein is extensively expressed in the cortex, hippocampus and cerebellum of wild type as compared with age-matched reeler mutants at P 30 (Fig 10B–10G).

## Discussion

Reelin has been described to control neuronal migration in the cerebral cortex, hippocampus and cerebellum during embryonic development [20–23, 59, 60]. In addition to these laminated brain structures, Reelin was also found to govern the lateral migration of some brainstem nuclei, such as midbrain dopaminergic nuclei [25–27] and of distinct groups of neurons in the spinal cord [61, 62]. After having shown that reelin signaling components are expressed in serotonergic neurons and that there is reelin expression nearby at E16.5–17.5, we studied a potential role of reelin signaling in the migration of brainstem serotonergic neuron development by analyzing mice deficient in reelin or Dab1 at different developmental time points.

We report that the migration of the rostral brainstem serotonergic neurons, in particular neurons of the paramedian raphe as well as of the lateral wings of the dorsal raphe, is indeed mediated by reelin-dab1 signaling. Disruption of reelin, the two reelin receptors ApoER2 and VLDLR or Dab1 resulted in an impairment of the lateral migration of serotonergic neurons destined to the paramedian raphe nuclei and to the lateral wings of the dorsal raphe nuclei. Several questions remain to be addressed in future studies. First, why are serotonergic neurons in the paramedian raphe and in the lateral wings of the dorsal raphe affected, whereas migration to the other raphe nuclei appeared virtually normal? Second, is the altered migration of these serotonergic neurons directly due to the deficiency of reelin, which in wild type animals might act as an attractive signal for the leading processes? Alternatively, is migration altered due to a malformation of a guiding fiber scaffold [63] in the absence of reelin signaling? Is reelin required for a change in migration directionality[28]? In the case of brainstem serotonergic neurons, reelin might be involved in the change from radial to lateral migration (present results). Our current and previous studies at least show that canonical reelin signaling is important for the normal positioning of mesencephalic dopaminergic neurons [25] and brainstem serotonergic raphe neurons, and the disruption of reelin signaling leads to an arrest in the lateral migration of the two affected neuronal populations. Of note, absence of reelin impaired the serotonergic projections to laminated brain structures, including hippocampus, neocortex, and cerebellum. We observed a dense projection of 5-HT positive fibers to all cortical layers in wild type. However, the serotonergic projections to the reeler cortex were clearly reduced and the prominent superficial and deep plexuses were not observed, as similarly reported by [64]. In the reeler hippocampus, we observed a massive reduction of 5-HT positive fibers, which may affect adult neurogenesis in the subgranular zone of the reeler dentate gyrus [65], since serotonin depletion is associated with a decrease of newly generated cells in the hippocampal dentate gyrus [66–68]. Moreover, it was reported reduction of the number of reelin-immunopositive cells in the dentate gyrus subgranular zone where adult hippocampal neurogenesis takes place in an animal model of depression induced by repeated corticosterone injections produces depression-like symptoms in both rats and mice that are reversed by antidepressant treatment [69]. Along this line, 5-HT positive fibers extend into the deep granular layer and Purkinje cell layer in wild type animals, in accordance with previous studies in the literature [70–72]. In contrast, 5-HT-positive terminals to the reeler cerebellum only reached the white matter and did not extend to the granular and molecular layers. It has been known since the discovery of reeler that lamination in the neocortex, hippocampus and cerebellum is severely affected in this mutant. Thus, the question arises, which needs to be addressed in future studies, whether the altered serotonergic innervation to these brain structures is due to the misplacement of serotonergic neurons or to the migration defects in their target structures resulting in the failure of the axons to find their appropriate destinations. In addition we show that serotonin transporter proteins (SERT) were highly reduced in laminated brain regions such as hippocampus, cortex, and cerebellum, which may explain the involvement of reelin in serotonergic neurons activity. The cortical neurons receive axonal projections from supralemniscal serotonergic cell group (B9) [73–76] rostral, ventral part and intrafascicular and dorsal parts of dorsal raphe nucleus [77–82] but not from lateral parts of dorsal raphe neurons, which project mainly to subcortical structures [83–85] and the contribution of B9 to the cortical innervation is probably much less than the 50% reduction in SERT, which explained that the remaining serotonin neurons on the midline may well have altered forebrain projections in reeler mice. [86] Mentioned that serotonergic signaling is most importantly regulated by the activity of SERT, which actually controls the concentration of active serotonin outside the cell. In summary then, we have described here a migration defect of brainstem serotonergic neurons in the reeler mouse, which adds to the severe migration defects of laminated brain structures in this mutant. Further studies need to address the functional consequences of this serotonergic neuron misplacement, given the widespread functions of the serotonergic system.

## Supporting information

S1 Fig Expression of vldlr receptors in the 5-HT positive neurons at E17.5. Scale bar: 50μm.
(TIF)

S2 Fig Expression of reelin in brainstem slices at E13.5 and E15.5. Scale bar: 300μm.
(TIF)
