# Correction: Reelin controls the positioning of brainstem serotonergic raphe neurons

**DOI:** 10.1371/journal.pone.0211849

**Published:** 2019-01-31

**Authors:** Reham Shehabeldin, David Lutz, Meliha Karsak, Michael Frotscher, Kerstin Krieglstein, Ahmed Sharaf

Fig 7 presents the 5-HT positive projections of the hindbrain serotonergic raphe neurons to the hippocampus in both wild type and reeler at postnatal day 20 (P20). The authors later discovered that the time point of used tissue was not from P20 but younger mice (P5). Please find the correct Fig 7 here, generated from brain tissue of WT and reeler mice of the correct age (P20).

**Fig 7:**
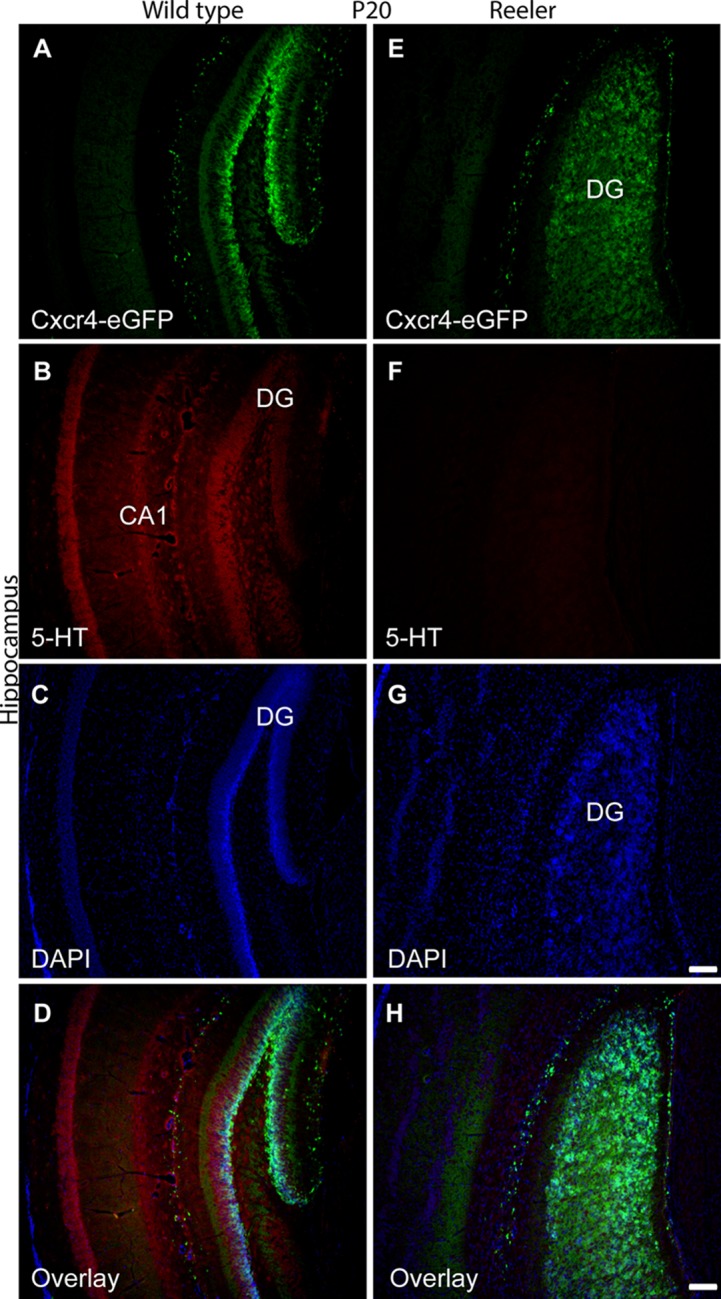
Altered serotonergic innervation of the reeler hippocampus at P20. (A) Expression of Cxcr4-eGFP in Cajal-Retzius (CR) cells of the dentate gyrus. (B-D) Serotonergic fibers are distributed throughout hippocampal layers, and 5-HT positive fibers and Cajal-Retzius cells overlap. (E) Scattered distribution of Cajal-Retzius cells in reeler hippocampus. (F-H) Severe reduction of serotonergic fibers in Cxcr4-eGFP hippocampal reeler mice. CA1, cornu ammonis area 1; CA3, cornu ammonis area 3; DG, dentate gyrus. Scale bar for A-D: 100μm.

In Fig 10, Fig 10H and Fig 10K were reversed accidentally. Fig 10H describes SERT positive fibers of serotonergic neurons into the hippocampus of reeler mice whereas Fig 10K describes SERT positive fibers of the serotonergic neurons into the hippocampus of WT mice. Please find the correct Fig 10 here, where the lower image (Fig 10K) has been be switched to the upper lane and vice versa.

**Fig 10:**
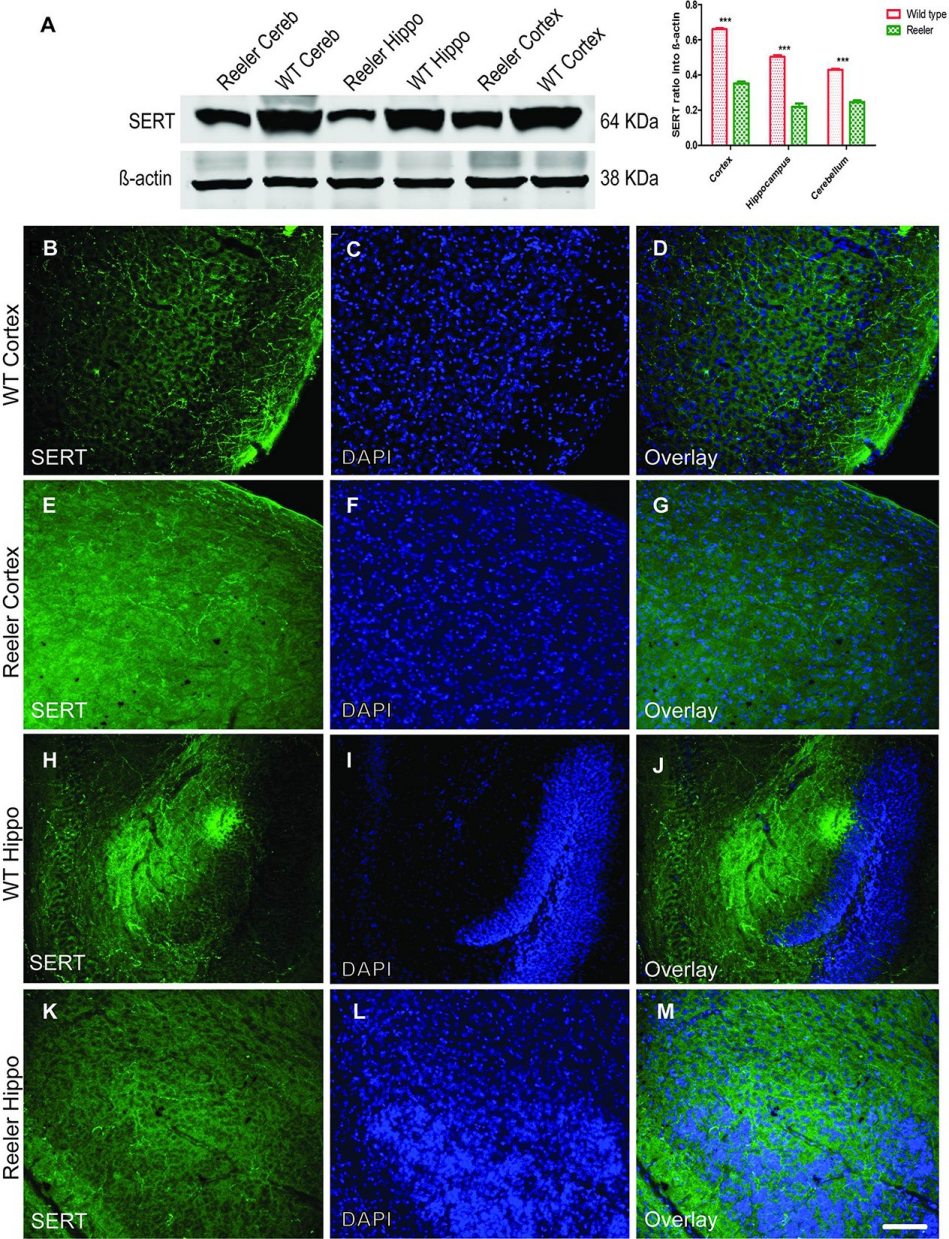
Reduction of serotonin transporter protein (SERT) expression in brain regions of reeler mice. A. Western blot analysis showed reduction of SERT protein levels in reeler cortex, hippocampus, and Cerebellum as compared to wild type. (B-G) reduction of SERT expression in reeler cortex as well as in hippocampal slices as observed in (H-M) as compared to the same matched wild type littermates at P30. Hippo = hippocampus; Cereb = Cerebellum. Scale bar: 100 μm.
