# Correction: Correction: Reelin controls the positioning of brainstem serotonergic raphe neurons

**DOI:** 10.1371/journal.pone.0213449

**Published:** 2019-02-28

**Authors:** The PLOS ONE Staff

There is an error in the Correction published on January 31, 2019. The publisher apologizes for this error.

The original Fig 7 caption was mistakenly included in the Correction, rather than the updated Fig 7 caption provided by the author. Please see the complete, correct Fig 7 caption here:

**Fig 7:**
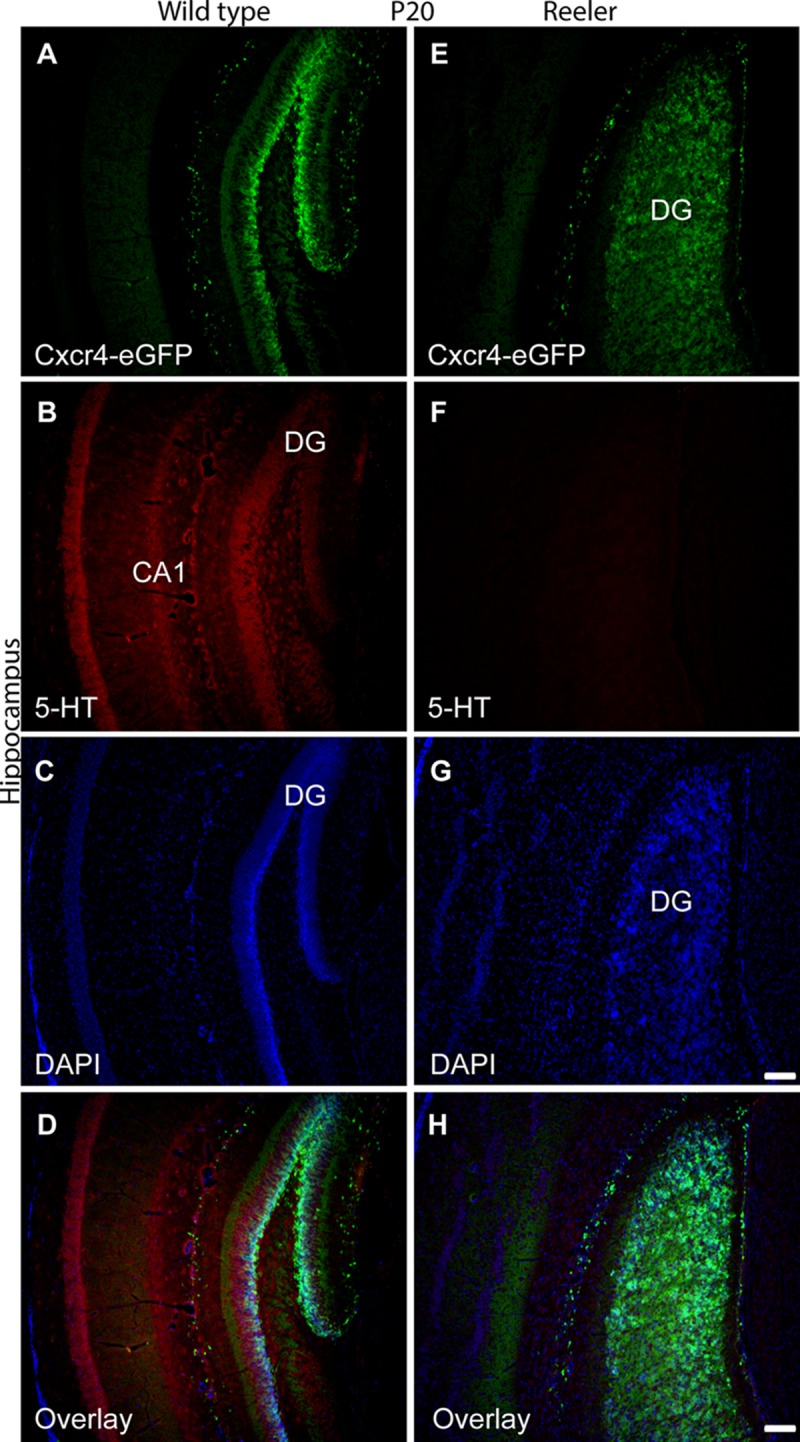
Altered serotonergic innervation of the reeler hippocampus at P20. (A) Expression of Cxcr4-eGFP (mouse anti-GFP antibody, 1:200, ab38689, Abcam) in Cajal-Retzius (CR) cells of the WT dentate gyrus (B-D) Serotonergic fibers are distributed throughout hippocampal layers. (E) Expression of Cxcr4-eGFP in CR cells in reeler hippocampus. (F-H) Severe reduction of serotonergic fibers in Cxcr4-eGFP hippocampal reeler mice. CA1, cornu ammonis area 1; DG, dentate gyrus. Scale bar for A-D: 100μm.

## References

[1] Shehabeldin R, Lutz D, Karsak M, Frotscher M, Krieglstein K, Sharaf A (2018) Reelin controls the positioning of brainstem serotonergic raphe neurons. PLoS ONE 13(7): e0200268 10.1371/journal.pone.0200268 30001399 PMC6042745

[2] Shehabeldin R, Lutz D, Karsak M, Frotscher M, Krieglstein K, Sharaf A (2019) Correction: Reelin controls the positioning of brainstem serotonergic raphe neurons. PLoS ONE 14(1): e0211849 10.1371/journal.pone.0211849 30703171 PMC6355025

