# Supplementary figures and images for: Reelin controls the positioning of brainstem serotonergic raphe neurons
Source: PLoS One. 2018 Jul 12;13(7):e0200268. doi: 10.1371/journal.pone.0200268 (PMC6042745; doi:10.1371/journal.pone.0200268)

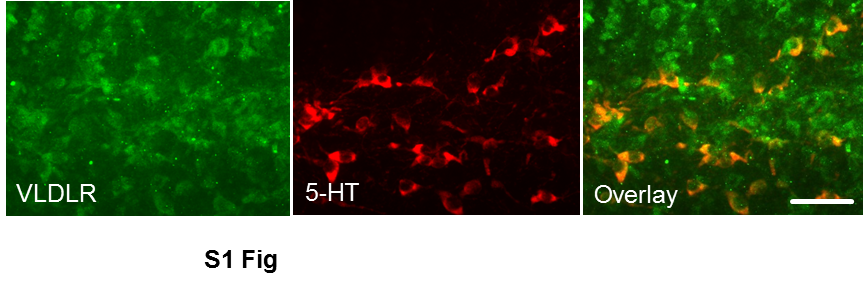

Supplement: S1 Fig — Scale bar: 50μm. (TIF) [file pone.0200268.s001.tif]

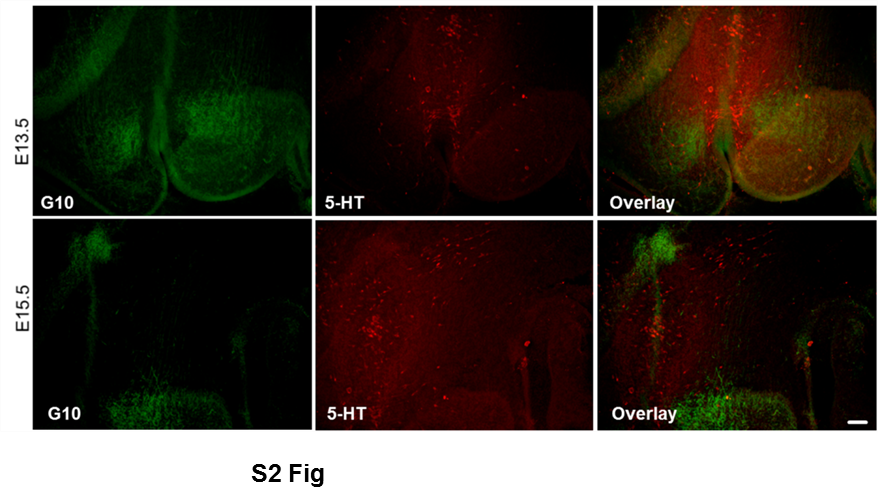

Supplement: S2 Fig — Scale bar: 300μm. (TIF) [file pone.0200268.s002.tif]
